# Thermoelectric characteristics of X$_2$YH$_2$ monolayers (X=Si, Ge; Y=P, As, Sb, Bi): a first-principles study

**DOI:** 10.1038/s41598-021-03280-1

**Published:** 2021-12-13

**Authors:** Mohammad Ali Mohebpour, Shobair Mohammadi Mozvashi, Sahar Izadi Vishkayi, Meysam Bagheri Tagani

**Affiliations:** 1 Computational Nanophysics Laboratory (CNL), Department of physics, University of Guilan, P. O. Box 41335-1914, Rasht, Iran; 2 School of Physics, Institute for Research in Fundamental Sciences (IPM), P. O. Box 19395-5531, Tehran, Iran

**Keywords:** Energy science and technology, Thermoelectric devices and materials

## Abstract

Ever since global warming emerged as a serious issue, the development of promising thermoelectric materials has been one of the main hot topics of material science. In this work, we provide an in-depth understanding of the thermoelectric properties of X$_2$YH$_2$ monolayers (X=Si, Ge; Y=P, As, Sb, Bi) using the density functional theory combined with the Boltzmann transport equation. The results indicate that the monolayers have very low lattice thermal conductivities in the range of 0.09−0.27 Wm$^{-1}$K$^{-1}$ at room temperature, which are correlated with the atomic masses of primitive cells. Ge$_2$PH$_2$ and Si$_2$SbH$_2$ possess the highest mobilities for hole (1894 cm$^2$V$^{-1}$s$^{-1}$) and electron (1629 cm$^2$V$^{-1}$s$^{-1}$), respectively. Si$_2$BiH$_2$ shows the largest room-temperature figure of merit, $ZT=2.85$ in the n-type doping ( $\sim 3\times 10^{12}$ cm$^{-2}$), which is predicted to reach 3.49 at 800 K. Additionally, Si$_2$SbH$_2$ and Si$_2$AsH$_2$ are found to have considerable *ZT* values above 2 at room temperature. Our findings suggest that the mentioned monolayers are more efficient than the traditional thermoelectric materials such as Bi$_2$Te$_3$ and stimulate experimental efforts for novel syntheses and applications.

## Introduction

Thermoelectric (TE) generators are considered as an eco-friendly solution to the global warming issue, since they can convert waste heat into electricity^1–5^. They have received considerable attention owing to their scalability, cleanliness, and long operating life^6–8^. A good TE material can improve the efficiency of photovoltaic^9–11^ and thermophotonic^12^ devices. The conversion efficiency of a TE material is measured by a dimensionless parameter called figure of merit (*ZT*)^13–15^ as below:

1 $$\begin{aligned} ZT=\frac{S^2\sigma T}{~\kappa _e+\kappa _L~}=\frac{PF}{~\kappa _e+\kappa _L~}T, \end{aligned}$$

where *S*, $\sigma$, and *T* are the Seebeck coefficient, electrical conductivity, and absolute temperature, while $\kappa _e$ and $\kappa _L$ stand for the electronic and lattice thermal conductivities, respectively. Generally, a promising TE material must have a large power factor $(PF=S^2\sigma )$ and low thermal conductivity $(\kappa =\kappa _e+\kappa _L)$. However, due to the complex correlations among the quantities, it is very difficult to achieve a large *ZT*.

Reduced dimensionality is regarded as an effective strategy for increasing the *ZT* of materials^16–20^, because it provides an opportunity to enhance the density of electronic states near the Fermi level, which subsequently increases the Seebeck coefficient. Also, it provides an opportunity to increase the charge carrier mobilities and relaxation times by decreasing the mean free paths. Most importantly, quantum confinement intensifies the boundary scattering of phonons at barrier-well interfaces, which leads to a reduction in the lattice thermal conductivity without increasing the electron scattering.

Two-dimensional (2D) materials have shown great potential in thermoelectric applications^21–23^. As reported by Zhang et al.^24^, a promising *ZT* value of $\sim$3.1 can be achieved by TiS$_3$ monolayer with a moderate carrier concentration at room temperature (300 K). Sang et al.^25^ showed that $\beta$-Te monolayer has very low lattice thermal conductivities (2.16 and 4.08 Wm$^{-1}$K$^{-1}$) and large *ZT*s (2.9 and 0.84) at 700 K for the armchair and zigzag directions, respectively. Moreover, Li et al.^26^ reported that InS, InSe, and InTe monolayers possess large *ZT*s (1.48, 1.74, and 2.03) at 300 K. In the case of InSe, Zeng et al.^27^ observed a substantial enhancement of the Seebeck coefficient and power factor by reducing the thickness and modulating the electron density. Furthermore, Bi$_2$Te$_3$-based materials have presented very good performances^2^. Recently, it was reported by Liu et al.^28^ that Bi$_{0.5}$Sb$_{1.5}$Te$_3$ nanomaterial could have a *ZT* of $\sim$1.96 at 420 K, which is higher than those of commercial materials.

Achieving a large *ZT* has been the main goal of the most TE researches. However, for practical applications, the toxicity and price of the materials should be taken into account. The aforementioned materials contain rare and toxic elements (S, Se, and Te), which diminish their actual applications. Therefore, the search for non-toxic and easy-to-prepare TE materials has remained a big challenge.

Very recently, a semiconducting monolayer, named Sn$_2$Bi, was synthesized on a silicon wafer, yielding a unique electronic structure and high chemical and thermal stability^29^. However, this monolayer is unstable without support of a substrate, which confines its applications in nano-scale devices. Subsequent theoretical works predicted that surface hydrogenation of Sn$_2$Bi can effectively stabilize the monolayer in free-standing form^30,31^. Additionally, it was predicted that fluorination not only can stabilize the free-standing Sn$_2$Bi but also leads to an ultralow lattice thermal conductivity of 0.19 Wm$^{-1}$K$^{-1}$ and an ultrahigh *ZT* value of 2.45 (1.70) at 300 K for n- (p-) type doping^32^.

Inspired by these results, in our previous work^33^, for the first time, we introduced and investigated a new class of 2D binary monolayers with an empirical formula of X$_2$Y, where X and Y are selected from group-IV (Si and Ge) and V (P, As, Sb, and Bi), respectively, including Si$_2$P, Si$_2$As, Si$_2$Sb, Si$_2$Bi, Ge$_2$P, Ge$_2$As, Ge$_2$Sb, and Ge$_2$Bi. We found that the pure X$_2$Y monolayers are unstable metals owing to the dangling bonds of X atoms. However, hydrogenation can effectively stabilize the monolayers by compensating the dangling bonds and fulfilling the octet rule. The hydrogenated X$_2$Y monolayers (X$_2$YH$_2$) are all semiconductors with band gaps predicted to be in the range of 1.17 to 2.39 eV. Besides, these monolayers have advantages such as earth abundance, environmental compatibility, and most importantly low toxicity, which make them very promising candidates for thermoelectric applications.

Herein, motivated by the amazing properties of X$_2$YH$_2$ monolayers, we investigate their thermoelectric properties. The results show that the monolayers have ultralow lattice thermal conductivities, which reflects the importance of the study. For instance, Si$_2$BiH$_2$ is found to have the largest room-temperature figure of merit $ZT=2.85$ in the n-type doping ( $\sim 3\times 10^{12}$ cm$^{-2}$) and is predicted to reach 3.49 at 800 K. Additionally, Si$_2$SbH$_2$ and Si$_2$AsH$_2$ are expected to show considerable *ZT* values of 2.73 and 2.02 at room temperature. Our work introduces a new class of thermoelectric materials which can be synthesized by a conventional process similar to the Sn$_2$BiH$_2$, as their constituent atoms belong to the same family and the former experimental work suggests possibility of similar syntheses^34^.

## Computational methods

The first-principles calculations were performed in the framework of density functional theory (DFT) using the Quantum ESPRESSO package^35^. The projector augmented wave (PAW) pseudopotential was used to describe the electron-ion interactions^36^. The generalized gradient approximation proposed by Perdew-Burke-Ernzerhof (GGA-PBE)^37^ was chosen to estimate the exchange-correlation potential. The energy cutoff was set to be 60 Ry. The Brillouin zone was sampled by a 11$\times$11$\times$1 k-point mesh. The energy convergence threshold for self-consistency was set to be $10^{-7}$ Ry. To eliminate the interactions coming from periodic boundary condition, a vacuum space of 20 Å was introduced along the z-direction. All structures were fully relaxed with a force tolerance of $10^{-3}$ eVÅ$^{-1}$. The total energy was converged with respect to the k-point mesh and energy cutoff to reach the threshold of $\sim$10$^{-6}$ Ry.

The electronic transport coefficients were obtained by solving the semiclassical Boltzmann transport equation (BTE) under the constant relaxation time approximation (CRTA), as implemented in BoltzTraP code^38^. The Boltzmann equation describes the change of carrier distribution function induced by external fields, lattice phonon scattering, or different kinds of defect scattering^39^. Due to the complexity of various carrier scattering mechanisms, it is almost impossible to obtain an exact solution of the Boltzmann equation. For simplicity, the relaxation time approximation is used. The Boltzmann method is widely used for the evaluation of transport properties of 2D materials and provides a good agreement with the experimental measurements^2^. In this method, the electronic band structure $\varepsilon$(k) is used to calculate the group velocity ($\nu _k$):

2 $$\begin{aligned} \nu _k=\frac{1}{\hbar }\frac{\partial \varepsilon (k)}{\partial k}. \end{aligned}$$

Then, the transport distribution function is determined from:

3 $$\begin{aligned} \Xi (\varepsilon )=\sum _k\nu _k\times \nu _k\tau _k, \end{aligned}$$

where $\tau _k$ is relaxation time at state *k*. Subsequently, the electrical conductivity ($\sigma$) and Seebeck coefficient (*S*) are respectively obtained by:^40^

4 $$\begin{aligned} \sigma (\mu ,T)= & {} e^2\int d\varepsilon \left( -\frac{\partial f_0(\varepsilon )}{\partial \varepsilon }\right) \Xi (\varepsilon ), \end{aligned}$$

5 $$\begin{aligned} S(\mu ,T)= & {} \frac{eK\!_B}{\sigma }\int d\varepsilon \left( -\frac{\partial f_0(\varepsilon )}{\partial \varepsilon }\right) \Xi (\varepsilon )\frac{\varepsilon -\mu }{K\!_BT}, \end{aligned}$$

where $f_0(\varepsilon )$ is the Fermi-Dirac distribution function, $\mu$ is the chemical potential, and $K_B$ is the Boltzmann constant. Also, the electronic thermal conductivity is calculated by the Wiedemann-Franz law given as, $\kappa _e=L\sigma T$, with *L* as the Lorenz number (2.45$\times$10$^{-8}$ W$\Omega$K$^{-2}$)^41^.

The thermal conductance ($\kappa _{ph}$) was calculated through:

6 $$\begin{aligned} \kappa _{ph}=\int \frac{~d\omega ~}{2\pi }~\hslash \omega ~T_{ph}(\omega )~\frac{\partial f_B(\omega ,T)}{\partial T}, \end{aligned}$$

where $\omega$ is the vibrational frequency, $f_B(\omega ,T)$ is the Bose-Einstein distribution function, and $T_{ph}(\omega )$ is the phonon transmission spectrum. In the ballistic regime, the transmission spectrum is obtained by the number of phonon bands crossing a particular energy. To capture an accurate spectrum, we employed a $300\times 300\times 1$ q-point grid. The Ballistic regime is widely used to calculate the lattice thermal conductivities of 2D materials and agrees very well with the experiments and theoretical works based on diffusive model^42,43^.

The phonon band structures were calculated to obtain the phonon transmission spectra of the monolayers. For this purpose, we firstly converted the hexagonal primitive cells into $\sqrt{3}\times 1\times 1$ rectangular cells and then repeated them into $5\times 5\times 1$ supercells containing 500 atoms. We employed the Fermi-Dirac smearing with a large width $\sigma = 0.5$ eV.

## Results and discussion

### Structural properties

**Figure 1:**
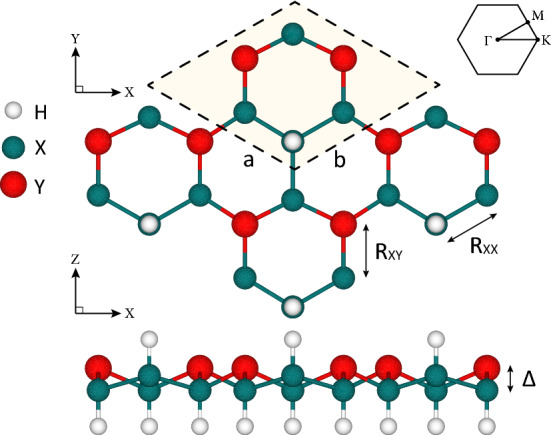
Top and side views of X$_2$YH$_2$ monolayers. The dark cyan balls show X (Si and Ge) atoms while the red balls show Y (P, Si, Sb, and Ge) atoms. Moreover, the white balls show hydrogen atoms. The unit cell and corresponding Brillouin zone are also illustrated.

**Figure 2:**
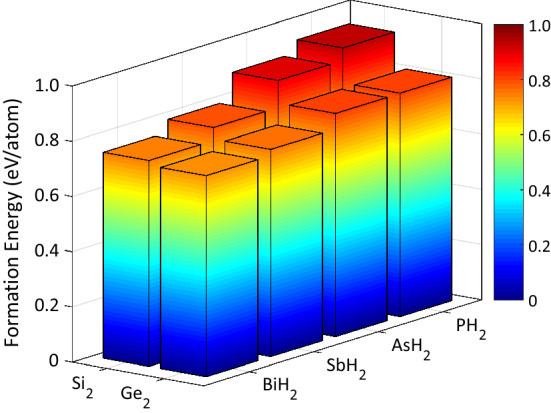
Formation energies of X$_2$YH$_2$ monolayers.

In our previous study^33^, it was discussed that to compensate the octet rule, X (Si and Ge) atoms should adsorb hydrogen. Different hydrogenation structures were considered. According to the cohesive energies, the double side hydrogenated model, having the lowest ground state energy, was predicted to be the most stable structure. Therefore, herein, we only focus on this hydrogenation model. Fig. 1 displays the top and side views of X$_2$YH$_2$ monolayers, where Xs are Si and Ge and Ys are P, As, Sb, and Bi. We call these hydrogenated monolayers Si$_2$PH$_2$, Si$_2$AsH$_2$, Si$_2$SbH$_2$, Si$_2$BiH$_2$, Ge$_2$PH$_2$, Ge$_2$AsH$_2$, Ge$_2$SbH$_2$, and Ge$_2$BiH$_2$. All the monolayers are optimized in hexagonal structure, such as the most stable group-IV and group-V 2D materials. After full relaxation, the lattice constants and buckling heights were respectively found to lie in the range of 6.26−7.18 and 1.08−1.38 Å, where the heavier atoms induce larger lattice constants and buckling heights. The optimized distance between the surface of Si (Ge) containing monolayers and H atoms was calculated to be 1.50 (1.56) Å. All the structural parameters are listed in Table 1. The structural, dynamical, and thermal stabilities of the monolayers were already validated by cohesive energy, phonon dispersion, and *ab-initio* molecular dynamics (AIMD) analyses in previous work^33^. We also calculated the formation energies $(E_f)$ through:

7 $$\begin{aligned} E_f=\frac{4E_X+2E_Y+2E_{H_2}-E_{sheet}}{10}, \end{aligned}$$

where $E_{sheet}$ is the total energy of monolayer, $E_X (E_Y)$ is the energy of a single atom *X*(*Y*) of the bulk structure, and $E_{H_2}$ is the ground state energy of a hydrogen molecule. The $E_X (E_Y)$ was obtained from a face-centered cubic (fcc) lattice known as stable phase. According to Eq. (7), it is obvious that positive formation energies is related exothermic chemical reactions, which implicit stable products. As listed in Table 1, the formation energies vary from 0.73 eV/atom for Ge$_2$BiH$_2$ to 0.94 eV/atom for Si$_2$PH$_2$, which indicates that all the monolayers are stable. Moreover, it is displayed in Fig. 2 that the stability is greater in structures with lighter atoms because they have higher tendency to form through an exothermic reaction.

**Table 1:** Structural parameters of X$_2$YH$_2$ monolayers including: lattice constants (*a*), bond lengths (*R*), buckling heights ($\Delta$), and formation energies ($E_f$).

	*a* (Å)	$R_{X\!X}$ (Å)	$R_{X\!Y}$ (Å)	$\Delta$ (Å)	$E_f$ (eV)
Si$_2$PH$_2$	6.26	2.35	2.27	1.08	0.94
Si$_2$AsH$_2$	6.44	2.35	2.39	1.19	0.89
Si$_2$SbH$_2$	6.79	2.35	2.60	1.30	0.79
Si$_2$BiH$_2$	6.94	2.35	2.69	1.35	0.75
Ge$_2$PH$_2$	6.52	2.46	2.36	1.15	0.81
Ge$_2$AsH$_2$	6.69	2.47	2.47	1.23	0.80
Ge$_2$SbH$_2$	7.03	2.47	2.67	1.33	0.75
Ge$_2$BiH$_2$	7.18	2.48	2.75	1.38	0.73

### Electronic properties

**Figure 3:**
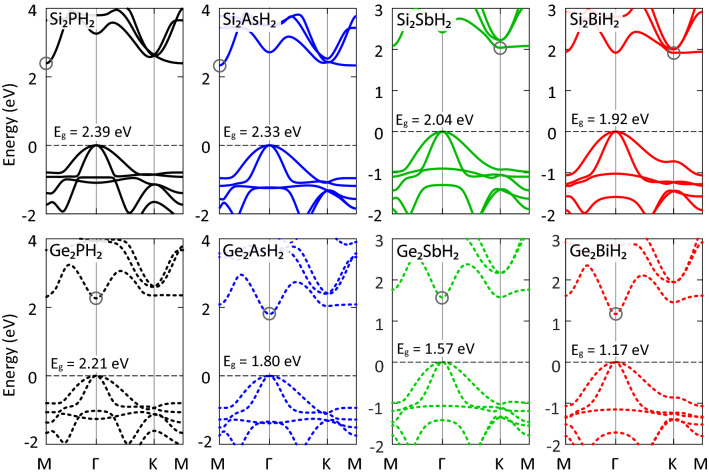
Band structures of X$_2$YH$_2$ monolayers. The VBMs were set to zero. The CBMs were specified with gray circles.

Investigation of thermoelectric features needs an adequate realization of electronic band gaps, carrier mobilities, and effective masses. As shown in the electronic band structures (Fig. 3), X$_2$YH$_2$ monolayers are semiconductors with band gaps predicted to be in the range of 1.17−2.39 eV. The band gap decreases monotonously with increasing the atomic mass. The Ge$_2$YH$_2$ monolayers have direct band gaps at the $\Gamma$ point. On the contrary, the Si$_2$YH$_2$ monolayers show indirect band gaps where the valence band maxima (VBM) are located at the $\Gamma$ point and the conduction band minima (CBM) are located at the M (Ge$_2$PH$_2$ and Ge$_2$AsH$_2$) and K (Ge$_2$SbH$_2$ and Ge$_2$BiH$_2$) points. In Si$_2$YH$_2$ monolayers, the CBMs are almost flat, suggesting strongly localized electrons with large effective masses. However, the VBMs are parabolically distributed, showing light holes.

In the conduction band of each monolayer, other than CBM, several relative dips are observed, which are called conduction band extrema (CBE). They provide a platform to achieve band convergence using mechanical strain, which further improves the thermoelectric properties^44^. In Si$_2$BiH$_2$ and Ge$_2$PH$_2$, the energy difference between the CBM and CBE is very small ($\sim 10^{-3}$ eV). Therefore, one can conclude that they have two CBMs, which are desirable for generating large transport coefficients in n-type doping. The overlap of the CBEs and CBM results in high transport of electrons without inter-valley scattering effect^45^.

We also checked the effects of spin-orbit coupling (SOC) in the band structures. It terminates the degeneracy of energy states and slightly reduces the band gaps. However, except for X$_2$BiH$_2$, the band gaps reduction is less than 0.1 eV. Hence, for its small influence on the electronic properties, the SOC is excluded from the TE calculations, except for the most efficient structure, which will found to be Si$_2$BiH$_2$.

### Thermoelectric properties

In the CRTA, the Seebeck coefficient is calculated independent of relaxation time ($\tau$). However, the electrical conductivity and the electronic thermal conductivity are obtained with respect to this parameter ($\sigma /\tau$, $\kappa _e/\tau$). Hence, we adopted the Bardeen and Shockley deformation potential theory^46^ to estimate the relaxation time from carrier mobility ($\mu$), considering the scattering between electrons and acoustic phonons as below:

8 $$\begin{aligned} \mu= & {} \frac{e\hslash ^3C^{2\mathrm {D}}}{~K\!_BTm^*m_dE^2_l~}, \end{aligned}$$

9 $$\begin{aligned} \tau= & {} \frac{\mu m^*}{e}, \end{aligned}$$

10 $$\begin{aligned} m^*= & {} \hslash ^2\left( \frac{d^2\varepsilon (k)}{dk^2} \right) ^{-1}, \end{aligned}$$

in which $C^{2\mathrm {D}}$ and $E_l$ stand for the in-plane elastic modulus and deformation potential, respectively. Also, $m^*$ and $m_d$ are the effective mass and average of effective mass defined by $m_d=\sqrt{m^*_xm^*_y~}$.

**Table 2:** Elastic constant ($C^{2\mathrm {D}}$), deformation potential ($E_l$), effective mass ($m^*$), carrier mobility ($\mu$), and relaxation time ($\tau$) for holes and electrons of X$_2$YH$_2$ monolayers. The mobility was calculated at 300 K along the zigzag and armchair directions.

	Carriers	$C^{2\mathrm {D}}$ (Jm$^{-2}$)	$E_l$ (eV)	$m^*$ ($m_0$)		$\mu$ (cm$^2$V$^{-1}$s$^{-1}$)		$\tau$ (fs)
				Zig	Arm	Zig	Arm	
Si$_2$PH$_2$	Hole	144.88	9.21	0.18	0.28	909.41	584.62	93.20
	Electron	144.88	6.95	2.30	0.10	55.50	1345.70	76.61
Si$_2$AsH$_2$	Hole	126.98	8.44	0.18	0.27	966.54	644.36	99.06
	Electron	126.98	7.28	2.95	0.12	29.37	722.02	49.33
Si$_2$SbH$_2$	Hole	108.49	8.03	0.16	0.20	1264.80	1011.80	115.21
	Electron	108.49	4.75	3.03	0.11	59.14	1629.10	102.03
Si$_2$BiH$_2$	Hole	91.43	10.79	0.25	0.13	374.91	720.97	53.37
	Electron	91.43	5.45	3.11	0.11	36.41	1029.41	64.48
Ge$_2$PH$_2$	Hole	122.42	7.58	0.44	0.11	473.58	1894.33	118.64
	Electron	122.42	23.01	0.13	0.12	306.39	331.92	22.68
Ge$_2$AsH$_2$	Hole	110.11	11.75	0.50	0.10	153.48	767.41	43.69
	Electron	110.11	21.01	0.10	0.10	536.70	536.70	30.56
Ge$_2$SbH$_2$	Hole	93.33	11.22	0.43	0.09	188.57	900.94	46.17
	Electron	93.33	20.06	0.08	0.07	833.55	952.64	37.97
Ge$_2$BiH$_2$	Hole	79.94	9.40	0.52	0.08	183.53	1193.11	54.34
	Electron	79.94	16.97	0.07	0.07	1218.90	1218.90	48.58

The elastic modulus and deformation potential are calculated by fitting processes using^47^

11 $$\begin{aligned} C^{2\mathrm {D}}= & {} \frac{2\partial ^2(E_\varepsilon -E_0)}{S_0\partial \varepsilon ^2}, \end{aligned}$$

12 $$\begin{aligned} E_l= & {} \frac{\Delta E}{\Delta a/a}, \end{aligned}$$

where $S_0$ is the surface of the unit cell, $E_\varepsilon$ and $E_0$ are the total energy at a small deformation state and equilibrium state, and $\Delta E$ is the variation of band edge (VBM and CBM) under lattice dilation $\Delta a/a$. This approach has been extensively adopted to determine the relaxation time of 2D materials^25,48,49^.

The calculated parameters are tabulated in Table 2. As can be seen, the elastic constant decreases with increasing the atomic mass of the monolayers. In other words, Si$_2$PH$_2$ and Ge$_2$BiH$_2$ possess the largest (144.88 Jm$^{-2}$) and the smallest (79.94 Jm$^{-2}$) elastic constants. This means that Ge$_2$BiH$_2$ is easier to change when the elastic deformation is applied. The softer structure induces a stronger electron scattering effect, which is detrimental for the electrical conductivity.

It is also found that the monolayers with strongly localized CBMs ( i.e. Si$_2$YH$_2$) have smaller deformation potential. This potential controls the scattering rate caused by electron-phonon interaction. Therefore, a smaller value of this constant can generate large carrier mobility. A higher deformation potential means that the electrons are more sensitive to the lattice perturbation (i.e. phonons).

In Si$_2$YH$_2$ monolayers, the effective mass of electron along the zigzag direction is relatively large, resulting from the flat CBMs along the K−M path, while for the armchair direction, it is very small in the range of 0.10 to 0.12 m$_0$. In Ge$_2$YH$_2$ monolayers, the effective mass of electron for both directions is very low in the range of 0.07 to 0.13 m$_0$. This is attributed to the parabolic CBMs. Meanwhile, the effective mass of hole along the armchair direction experiences a decreasing trend with increasing the atomic mass. However, there is no specific order for the zigzag direction.

Using all these quantities, we calculated the carrier mobilities of X$_2$YH$_2$ monolayers along both directions as listed in Table 2. Obviously, there is a strong anisotropy which is dominated by the corresponding anisotropy of effective mass. For electrons, the mobility along the armchair is larger than that of the zigzag direction, while for holes, there is no specific order. Regardless of direction, the highest mobility for holes (1894 cm$^2$V$^{-1}$s$^{-1}$) and electrons (1629 cm$^2$V$^{-1}$s$^{-1}$) belong to Ge$_2$PH$_2$ and Si$_2$SbH$_2$, respectively. Compared to the mobilities reported for SnS (623), SnSe (1035), GeS (1045), GeSe (541), Te (1343), ZrS$_2$ (1045), and MoS$_2$ (200 cm$^2$V$^{-1}$s$^{-1}$)^25,48–51^, Ge$_2$PH$_2$ and Si$_2$SbH$_2$ are promising candidates for high-speed nanoelectronic devices. Moreover, one can conclude that Si$_2$SbH$_2$, Si$_2$BiH$_2$, and Ge$_2$BiH$_2$ are favorable for field-effect transistors owing to their high mobilities for both carriers.

**Figure 4:**
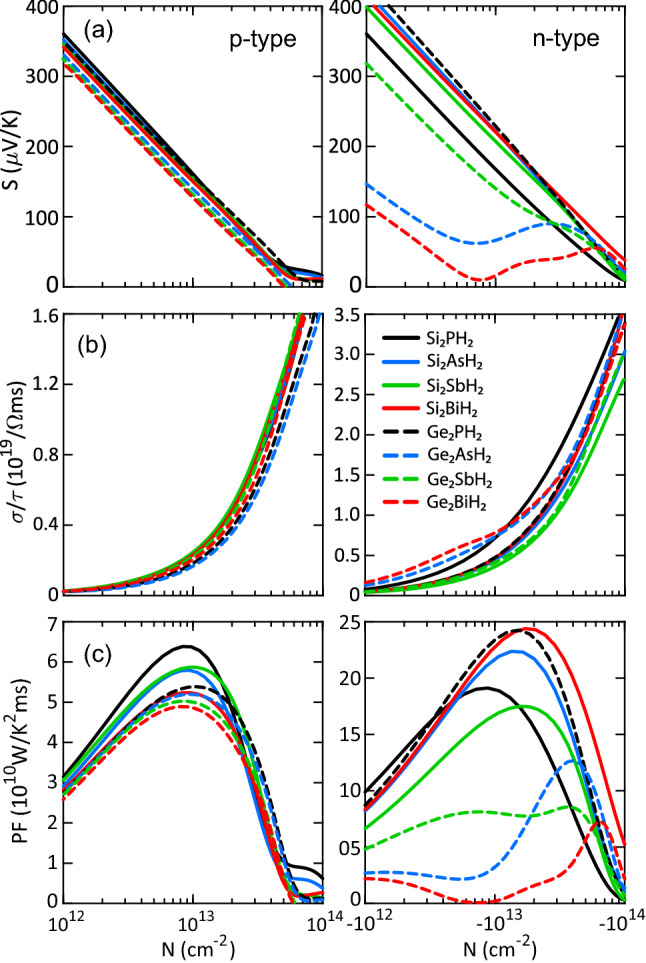
Electronic transport coefficients of X$_2$YH$_2$ monolayers including the (**a**) Seebeck coefficient, (**b**) electrical conductivity, and (**c**) power factor as a function of carrier concentration at 300 K. The Seebeck coefficient of n-type doping was reversed to positive values for simplicity. The carrier concentration denotes the numbers of electrons or holes per the surface of the unit cell.

After discussing the structural and electronic characteristics of X$_2$YH$_2$ monolayers, now we have sufficient information and insights to concentrate on the thermoelectric properties. Fig. 4 represents the transport coefficients of X$_2$YH$_2$ monolayers. Within the framework of the rigid band model, the results are evaluated for p- and n-type doping, so that the types of doping are mimicked by shifting the Fermi level into the valence and conduction bands, respectively. As it is clear, Ge$_2$BiH$_2$ has the lowest Seebeck coefficient in both doping types, because it has the smallest band gap (1.17 eV). The maximum values obtained for this monolayer are 1673 and 1629 $\mu$VK$^{-1}$ for p- and n-type doping, respectively. By increasing the band gap, the Seebeck coefficient is expected to increase. Hence, Si$_2$PH$_2$ has the highest value (2757 $\mu$VK$^{-1}$) in the p-type doping. In the n-type doping, Ge$_2$PH$_2$ shows the highest (2832 $\mu$VK$^{-1}$) peak because the Seebeck coefficient is inversely dependent on the effective mass, and the effective mass of electrons for Si$_2$PH$_2$ is very large compared to that of Ge$_2$PH$_2$ (see Table 2).

No considerable differences are observed in the p-type electrical conductivities. This is probably due to the almost identical valence bands (see Fig. 3). In the n-type doping, it depends on the carrier concentration. At a low level of doping, the highest electrical conductivity belongs to Ge$_2$BiH$_2$ because it has a very low Seebeck coefficient at this range. Also, it is found that the n-type electrical conductivities are larger than those of the p-type ones, which is attributed to the presence of several extrema in the conduction bands. The extrema can provide more electrons to participate in transport.

The Seebeck coefficient reaches its maximum value at a low level of carrier concentration. But at this level, the electrical conductivity is very small. To optimize the concentration, we calculated the power factor (*PF*). As presented in Fig. 4(c), Si$_2$PH$_2$ produces the largest power factor (6.47$\times$10$^{10}$ WK$^{-2}$m$^{-1}$s$^{-1}$) in the p-type doping ($\sim$9$\times$10$^{12}$ cm$^{-2}$), resulting from the moderate electrical conductivity and large Seebeck coefficient. Including the corresponding relaxation time (93.20$\times$10$^{-15}$ s), it becomes $6.03\times 10^{-3}$ WK$^{-2}$m$^{-1}$. In a higher level of p-type doping ($\sim$2.3$\times$10$^{15}$ cm$^{-2}$), the power factor of Si$_2$PH$_2$ reaches 16.94$\times$10$^{10}$ WK$^{-2}$m$^{-1}$s$^{-1}$. However, due to the direct proportion between doping level and thermal conductivity, it will not lead to larger figure of merit. On the other hand, Si$_2$BiH$_2$ and Ge$_2$PH$_2$ have the largest power factor ($\sim$24.3$\times$10$^{10}$ WK$^{-2}$m$^{-1}$s$^{-1}$) in the n-type doping ($\sim$2$\times$10$^{13}$ cm$^{-2}$). Including the corresponding relaxation times, it becomes 15.66 and $5.51\times 10^{-3}$ WK$^{-2}$m$^{-1}$ for Si$_2$BiH$_2$ and Ge$_2$PH$_2$, respectively. Overall, one can say that the n-type doping offers much better thermoelectric performance than the p-type one.

**Figure 5:**
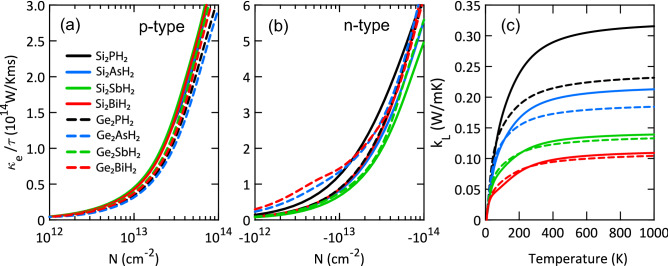
Electronic thermal conductivity of X$_2$YH$_2$ monolayers for (**a**) p- and (**b**) n-type doping at 300 K together with their (**c**) lattice thermal conductivities as a function of temperature.

Figure 5 indicates the thermal conductivities of X$_2$YH$_2$ monolayers. As can be seen, the electronic thermal conductivities are almost similar to the electrical conductivities, because they are connected through the Wiedemann-Franz law given as, $\kappa _e=L\sigma T$^41^. The lattice thermal conductivity follows the expected trend with increasing the atomic mass. More specifically, Si$_2$BiH$_2$ and Ge$_2$BiH$_2$ have the lowest thermal conductivity of 0.09 Wm$^{-1}$K$^{-1}$ at 300 K. This is due to their lower Debye temperature, smaller phonon group velocity, and stronger anharmonicity compared to other monolayers as discussed in the previous work^33^. Also, Si$_2$SbH$_2$ and Ge$_2$SbH$_2$ exhibit very low thermal conductivity of 0.12 Wm$^{-1}$K$^{-1}$. Such low lattice thermal conductivities originate from large buckling heights because flexural phonons have more scattering channels in buckled structures and consequently less contribution to heat transport. The calculated values are smaller than those of $\beta$-Bi (3.8)^52^, Bi$_2$Te$_3$ (1.1)^53^, MoS$_2$ (1.03), MoSe$_2$ (0.72), MoTe$_2$ (0.54), WS$_2$ (0.83), WSe$_2$ (0.66), WTe$_2$ (0.50), TiS$_2$ (0.95), TiSe$_2$ (0.95), and TiTe$_2$ (0.70 Wm$^{-1}$K$^{-1}$)^54^, making them potential candidates for thermoelectric applications. Figure 5(c) also shows that the lattice thermal conductivities of X$_2$YH$_2$ are gradually saturated and tend to constant values. This is due to the increase of phonon scattering at high temperatures, which is stronger in heavier structures.

Phonon band structures and transmission coefficients of X$_2$YH$_2$ monolayers are given in Figure S1 and Figure S2, respectively. The results reveal a direct relationship between the atomic mass of the unit cell and the gap observed in the phonon dispersion. More specifically, increasing the atomic mass increases the phonon band gap observed in the optical modes and reduces the band linewidth. The presence of band gap and dispersionless nature of the phonon modes are key factors to reduce the thermal conductivity, very important to obtain high thermoelectric efficiency.

The results also manifest that the thermal conductivities of the monolayers strongly depend on the atomic masses of constituent elements and the temperature. At high temperatures, Si$_2$YH$_2$ monolayers have higher thermal conductivities than Ge$_2$YH$_2$ ones. Importantly, the critical temperature, the temperature at which the thermal conductivities of Si$_2$YH$_2$ exceeds those of Ge$_2$YH$_2$, increases with increasing the atomic mass of element Y. Figure S3 shows the variation of critical temperature with element Y.

According to Eq. 6, one can simply show that the thermal conductance for low energies ($\hslash \omega<<k_BT$) can be obtained through:

13 $$\begin{aligned} \kappa _{ph}\approx K_B\int \frac{~d\omega ~}{2\pi }~T_{ph}(\omega ). \end{aligned}$$

At low energies, acoustic phonons play the main role in heat transport. As can be seen from the phonon band structures, the out-of-plane flexural acoustic mode (ZA) has parabolic behavior near the $\Gamma$ point while the in-plane longitudinal and transverse acoustic phonon modes (LA and TA) behave linearly. For a better understanding of the thermal conductivity of phonons, we fitted the ZA, LA, and TA modes with $\alpha _z q^2$, $\alpha _L q$, and $\alpha _T q$ functions, respectively. The final results listed in Table S1 shows that with decreasing the atomic mass of element Y, $\alpha _z$ increases linearly. The gradient of increase is higher in Si$_2$YH$_2$. This difference between the gradients leads to a considerable gap between thermal conductivity of Si$_2$YH$_2$ and Ge$_2$YH$_2$ monolayers. This gap increases with increasing temperature and decreasing the atomic mass of element Y. Also, $\alpha _L$ and $\alpha _T$ increase with decreasing the atomic mass of element Y.

**Figure 6:**
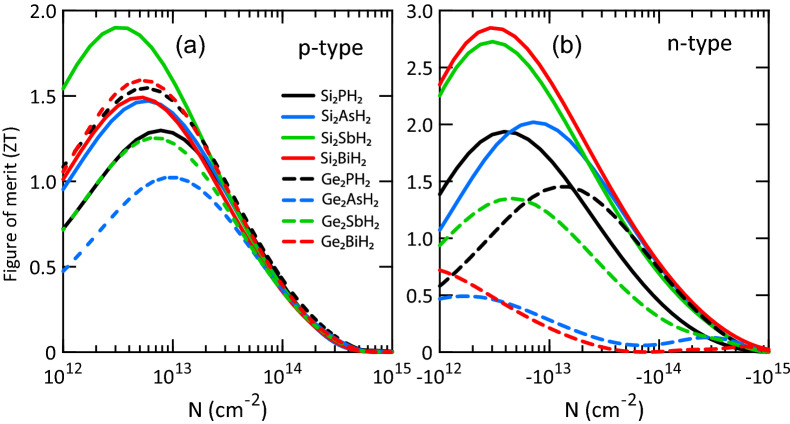
Thermoelectric figure of merit (*ZT*) of X$_2$YH$_2$ monolayers for (**a**) p- and (**b**) n-type doping at 300 K.

At high energies $(\hbar \omega>>K_BT)$, the thermal conductance is obtained by:

14 $$\begin{aligned} \kappa _{ph}\approx \frac{1}{K_BT^2}\int ~d\omega ~(\hslash \omega )^2~T_{ph}(\omega ), \end{aligned}$$

where, in addition to the acoustic phonons, the optical phonons are also excited and contribute to carrying energy. Here, the reason for the lower thermal conductivity of heavier structures directly lays behind the energy gap emerged in their optical phonons. As it is clear in the phonon band structures (Figure S1) and the phonon transport coefficients (Figure S3), with increasing the atomic mass of element Y, the band gap between optical modes increases. The energy gap between phonon modes stops the energy transport. The gap has a direct correlation with the mass of element Y. According to the obtained results, it is obvious that Ge$_2$BiH$_2$ monolayer has the lowest thermal conductivity because it has the heaviest Y atom, and on the other hand, Ge has smaller phonon modes in comparison to Si. Another important achievement of this investigation is that the atomic mass of element Y is way more important than that of element X, so that, Si$_2$BiH$_2$ has lower thermal conductivity than Ge$_2$SbH$_2$.

To ensure the accuracy of the results, we calculated the lattice thermal conductivity of black phosphorene. At 300 K, it was obtained as 0.57 and 0.39 Wm$^{-1}$K$^{-1}$ for the zigzag and armchair directions, respectively, which are in a great agreement with the values reported by Sevik et al. (0.55 and 0.35 Wm$^{-1}$K$^{-1}$)^55^. Also, we calculated the lattice thermal conductivity of the hydrogenated Sn$_2$Bi (Sn$_2$BiH$_2$) monolayer as 0.51 Wm$^{-1}$K$^{-1}$. This is only 26% lower than the previous value (0.69 Wm$^{-1}$K$^{-1}$) calculated considering the phonon-phonon scattering^32^.

Using the electronic transport coefficients and lattice thermal conductivities, we calculated the *ZT* values of X$_2$YH$_2$ monolayers at 300 K as illustrated in Fig. 6. In the p-type doping, the largest *ZT* (1.90) is realized by Si$_2$SbH$_2$ at the carrier concentration of $3\times 10^{12}$ cm$^{-2}$, where the Seebeck coefficient is $\sim$280 $\mu$VK$^{-1}$. The corresponding electronic thermal conductivity is 1.57 Wm$^{-1}$K$^{-1}$, which is nearly 13 times larger than the lattice thermal conductivity (0.12 Wm$^{-1}$K$^{-1}$). Indeed, one can say that the holes play a dominant role in the total thermal conductivities. On the contrary, in the n-type doping, Si$_2$BiH$_2$ (2.85) and Si$_2$SbH$_2$ (2.73) produce the largest *ZT*s. The peaks are found at the carrier concentration of $3\times 10^{12}$ cm$^{-2}$, where the Seebeck coefficients are $\sim$320 and 310 $\mu$VK$^{-1}$ and the electronic thermal conductivities are 1.01 and 1.73 Wm$^{-1}$K$^{-1}$, respectively. This shows the dominant contribution of electrons in the thermal conductivities, although they might be a bit overestimated. Also, the maximum *ZT* obtained for Si$_2$ASH$_2$ and Si$_2$PH$_2$ are 2.02 and 1.94, respectively, which exceed the standard of applicable TE materials. Thus, Si$_2$YH$_2$ monolayers are obviously more efficient than the traditional TE materials such as Bi$_2$Te$_3$^53,54^. Their excellent performances are attributed to their large power factors and ultralow lattice thermal conductivities.

**Figure 7:**
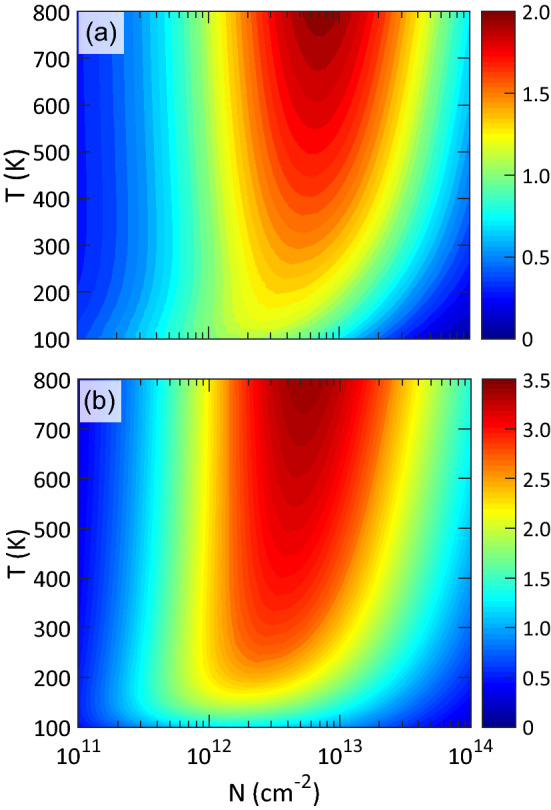
Two-dimensional contour plot of *ZT* values for (**a**) p- and (**b**) n-type Si$_2$BiH$_2$ vs temperature and carrier concentration. The dark blue (red) shows the lowest (highest) value of *ZT*.

For Si$_2$BiH$_2$, the dependence of *ZT* values on temperature is investigated. As can be seen from Fig. 7, by increasing the temperature, the *ZT* values increase gradually and the corresponding peaks shift to the higher levels of carrier concentration. For instance, at 500 and 800 K, the *ZT* reaches 3.16 (1.75) and 3.49 (2.04) for n- (p- ) type doping, respectively, as the optimal carrier concentration approaches $10^{13}$ cm$^{-2}$. This behavior is due to the increase of electrical conductivity with temperature, while the lattice thermal conductivity remains almost constant. The results indicate that Si$_2$BiH$_2$ monolayer is capable of working effectively in a wide range of temperature.

As discussed earlier, negative spin-orbit strength reduces the band gap of structures. Therefore, in the presence of SOC, the Seebeck coefficient is expected to decrease while the electrical conductivity is likely to increase. Here, we check the effects of SOC on the *ZT* values of Si$_2$BiH$_2$. Our results reveal that the maximum *ZT* is 1.77 and 3.14 for p- and n-type doping, respectively. Compared to those without SOC ( 1.49 and 2.85), one can say that spin-orbit interaction improves the p- and n-type TE performance by almost 18% and 10%, respectively. For other structures, the inclusion of SOC may give rise to quantitative changes in the thermoelectric coefficients. However, owing to its negligible impact, it is neglected. The same behavior was reported for the Sn$_2$BiH$_2$ monolayer, where the inclusion of SOC increases the peak of power factor by 20%^44^.

Very recently, the pure and hydrogenated Sn$_2$Bi was synthesized by chemical vapor deposition (CVD)^29,34^. Therefore, a similar process may be used to synthesize X$_2$YH$_2$ monolayers. For instance, to prepare the Si$_2$BiH$_2$, more than 1 monolayer (ML) of high-purity Bi gas is grown on a substrate. After annealing, in second step, Si atoms are deposited on the surface to form the honeycomb Si$_2$Bi. For hydrogenation, the sample should be in the exposure of hydrogen gas. Different techniques including the Birch reaction, high-pressure hydrogenation, H-plasma procedure, and poly-amine hydrogenation can be implemented to hydrogenate Si$_2$Bi as they worked well for graphene^56^. ZnS (111), SiC (111), and Si (111) insulators can be used as the substrate.

## Conclusion

Summarily, we used density functional theory combined with the Boltzmann transport equation to evaluate the thermoelectric properties of X$_2$YH$_2$ monolayers (X=Si, Ge; Y=P, As, Sb, Bi). The results manifest that the monolayers have very low lattice thermal conductivities at room temperature, which are associated with the atomic masses of primitive cells. Also, it is found that the n-type doping offers much better thermoelectric performance than the p-type one. Si$_2$BiH$_2$ has the largest room-temperature figure of merit, $ZT=2.85$ in the n-type doping ( $\sim 3\times 10^{12}$ cm$^{-2}$) and is predicted to reach 3.49 at 800 K. Spin-orbit coupling improves the thermoelectric performance by almost 10%. Besides, Si$_2$SbH$_2$ and Si$_2$AsH$_2$ show relatively large *ZT*s of 2.73 and 2.02, respectively. Such large *ZT*s suggest that the monolayers could be excellent thermoelectric materials. Due to the abundance and non-toxicity of the constituent elements, Si$_2$YH$_2$ are good candidates for thermoelectric applications and deserve much attention in the experimental field.

## Supplementary Information

Supplementary Information.
