# Supplementary material for: Thermoelectric characteristics of X2YH2 monolayers (X=Si, Ge; Y=P, As, Sb, Bi): a first-principles study
Source: Sci Rep. 2021 Dec 13;11:23840. doi: 10.1038/s41598-021-03280-1 (PMC8668932; doi:10.1038/s41598-021-03280-1)
Supplement: Supplementary file 1 — Supplementary Information. [file 41598_2021_3280_MOESM1_ESM.pdf]

Supporting Information for

Thermoelectric Characteristics of X_2YH_2 Monolayers (X=Si, Ge; Y=P, As, Sb, Bi): A First-Principles Study

Mohammad Ali Mohebpour[1], Shobair Mohammadi[1], Sahar Izadi Vishkayi[2], Meysam Bagheri Tagani[1]*,

[1]*Computational Nanophysics Laboratory (CNL), Department of Physics, University of Guilan, P. O. Box 41335-1914, Rasht, Iran.*

[2]*School of Physics, Institute for Research in Fundamental Sciences (IPM), P. O. Box 19395-5531, Tehran, Iran.*

Corresponding Author. Email: m_bagheri@guilan.ac.ir

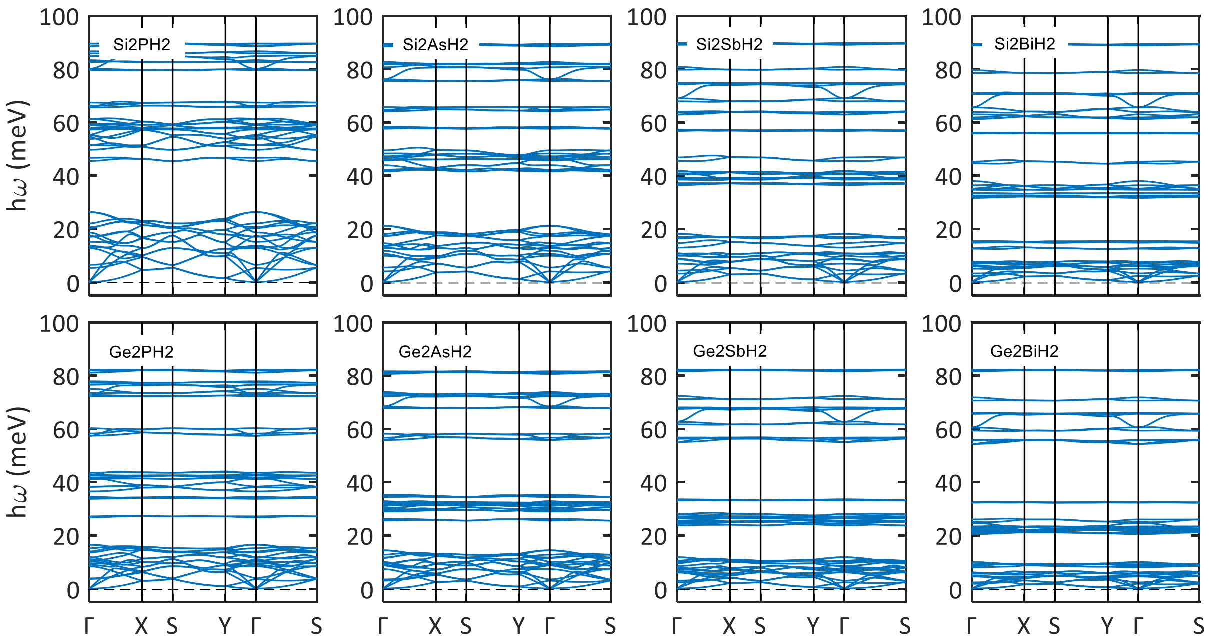

Figure S1. Phonon dispersion spectra of the hydrogenated X_2Y monolayers.

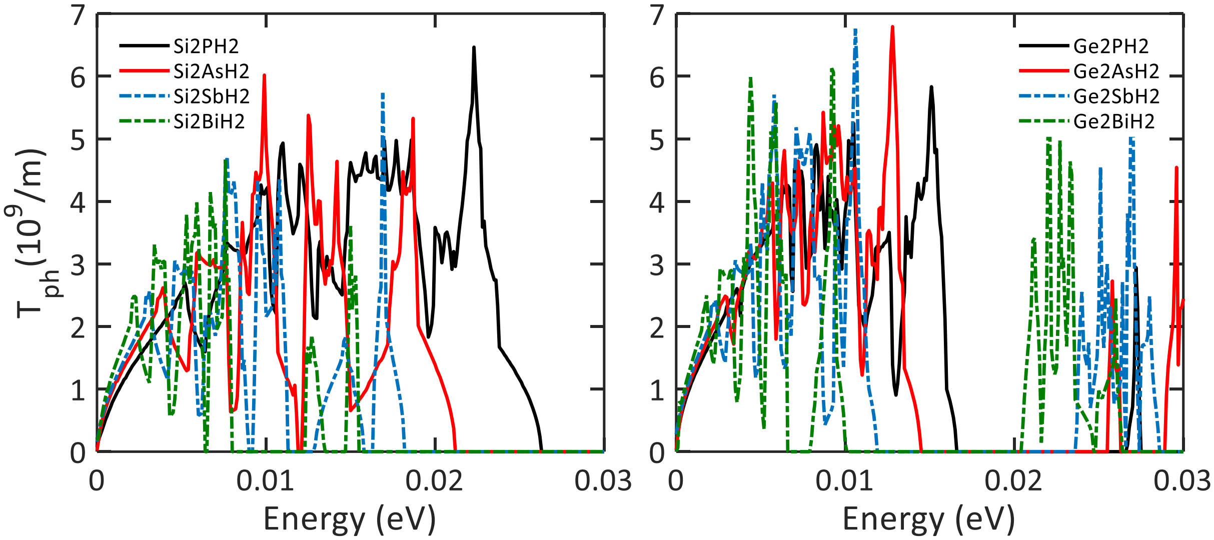

Figure S2. Phonon transmission coefficients of the hydrogenated X_2Y monolayers.

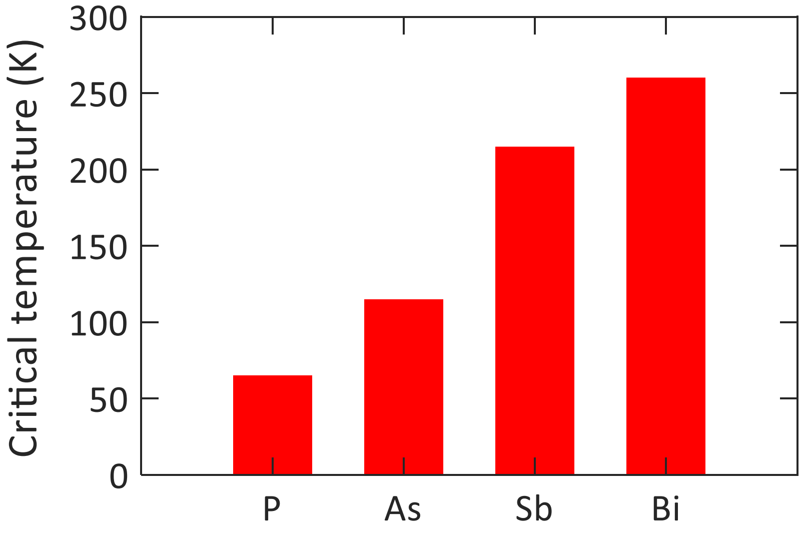

Figure S3. Variation of critical temperature with element Y (P, As, Sb, and Bi).

Table S1. Parameters achieved from fitting the acoustic phonon modes of ZA, LA, and TA with $\alpha_Z q^2$, $\alpha_L q$, and $\alpha_T q$ functions, respectively.

Monolayer	α_z (meV/Å^2)	α_L (meV/Å)	α_T (meV/Å)
Si_2PH_2	81.84	21.40	31.26
Si_2AsH_2	64.95	16.75	23.22
Si_2SbH_2	49.75	12.95	17.13
Si_2BiH_2	35.73	9.00	12.00
Ge_2PH_2	52.76	14.09	20.46
Ge_2AsH_2	47.46	11.77	17.47
Ge_2SbH_2	38.69	9.50	13.45
Ge_2BiH_2	29.62	7.07	9.84
